# Complications after endovascular stent-grafting of thoracic aortic diseases

**DOI:** 10.1186/1749-8090-1-26

**Published:** 2006-09-12

**Authors:** Gabriele Piffaretti, Matteo Tozzi, Chiara Lomazzi, Nicola Rivolta, Roberto Caronno, Patrizio Castelli

**Affiliations:** 1 Vascular Surgery-Department of Surgery, University of Insubria-Varese, Italy

## Abstract

**Background:**

To update our experience with thoracic aortic stent-graft treatment over a 5-year period, with special consideration for the occurrence and management of complications.

**Methods:**

From December 2000 to June 2006, 52 patients with thoracic aortic pathologies underwent endovascular repair; there were 43 males (83%) and 9 females, mean age 63 ± 19 years (range 17–87). Fourteen patients (27%) were treated for degenerative thoracic aortic aneurysm, 12 patients (24%) for penetrating aortic ulcer, 8 patients (15%) for blunt traumatic injury, 7 patients (13%) for acute type B dissection, 6 patients (11%) for a type B dissecting aneurysm; 5 patients (10%) with thoraco-abdominal aortic aneurysms were excluded from the analyses. Fifteen patients (32%) underwent emergency treatment. Overall, mean EuroSCORE was 9 ± 3 (median 15, range 3–19). All procedures were performed in the theatre under general anesthesia. All complications occurring during hospitalisation were recorded. Follow-up protocol featured CT-A, and chest X-rays 1, 4 and 12 months after intervention, and annually thereafter.

**Results:**

Primary technical success was achieved in all patients; procedures never aborted because of access difficulty. Conversion to standard open repair was never required. Mean duration of the procedure was 119 ± 75 minutes (median 90, range 45–285). Mean blood loss was 254 mL (range 50–1200 mL). The mean length of the aorta covered by the SGs was 192 ± 21 mm (range 100–360). The LSA was over-stented in 17 cases (17/47, 36%). Overall 30-day operative mortality was 6.4% (3/47). Major complications included pneumonia (n = 9), cerebrovascular accidents (n = 4), arrhythmia (n = 4), acute renal failure (n = 3), and colic ischemia (n = 1). Overall, endoleak rate was 14%.

**Conclusion:**

Although this report is a retrospective and not comparative analysis of thoracic aortic repair, the combined minor and major morbidity rate was lower than previous reported to results of either electively and emergency performed conventional repair.

## Background

Endovascular treatment for aortic disease has emerged as an alternative mode of treatment for thoracic aortic diseases (TADs) that is particularly attractive for patients with severe co-morbidities who would not be ideal candidates for open surgery [1-6]. Because initial results have been encouraging, this new therapy has been used more liberally, and short-term results are well documented [1,4,5] However, despite early promising clinical results, effect on the long-term durability of the aortic repair have not been yet established, they are associated with complications, as are all surgical methods [7-9].

Here, we present our experience with TAA stent-graft (SG) treatment, with special consideration for the occurrence and management of complications.

## Materials and methods

From December 2000 to June 2006, 52 patients with thoracic aortic pathologies underwent endovascular repair; there were 43 males (83%) and 9 females, mean age 62 ± 19 years (range 17–87). Fourteen patients (27%) were treated for degenerative thoracic aortic aneurysm, 12 patients (24%) for penetrating aortic ulcer, 8 patients (15%) for blunt traumatic injury, 7 patients (13%) for acute type B dissection, 6 patients (11%) for a type B dissecting aneurysm. Five additional patients who received hybrid treatment (visceral revascularization and endovascular thoracic SG) for thoraco-abdominal aortic aneurysms were excluded from this series. Hence, 47 patients wree considered for the retrospective analyses. Patient data including procedure details, postoperative complications, secondary procedures, and mortality were collected. Demographics and co-morbidities are listed in Table 1.

**Table 1:** Demographic data and co-morbidities

*disease*	*nr*	*%*
hypertension	34	72
obesity (BMI > 30)	21	45
COPD	19	40
IHD	13	28
CRF	8	17
previous aortic surgery	7	15
hypercholesterolemia	5	10
previous cardiovascular surgery	5	10
CVD	4	8
diabetes mellitus	4	8

BMI = body mass index; COPD = chronic obstructive pulmonary disease; IHD = ischemic heart disease CRF = chronic renal failure; CVD = cerebrovascular disease

Fifteen patients (32%) underwent emergency treatment because of blunt traumatic injury (n = 7), complicated dissection (n = 4), penetrating ulcer (n = 3), or ruptured degenerative aneurysm. Eight patients (17%) had infrarenal aortic aneurysm repair in their medical history.

The Ishimaru classification were used to define the location of the proximal neck of the lesion: "zone 0" involves the innominate artery, "zone 1" the origin of the left common carotid artery, "zone 2" the origin of the left subclavian artery, "zone 3" extends into the descending thoracic aorta distally to the origin of the left subclavian artery, "zone 4" involved the middle to the lower third of the descending aorta: according to the Ishimaru classification, the aortic pathology was located in the aortic arch (zone 1–2) in 12 patients, in proximal/mid-descending aorta (zone 3) in 25 patients, and in the distal descending aorta (zone 4) in 10 patients. Mean thoracic aortic aneurysm diameter was 7.5 ± 2 cm (range 5.5–12), mean dissecting aneurysm diameter was 7.3 ± 2 cm (range 6–9), and mean ulcer diameter was 4.3 ± 0.8 cm (range 3.5–6.5).

**Figure 1:**
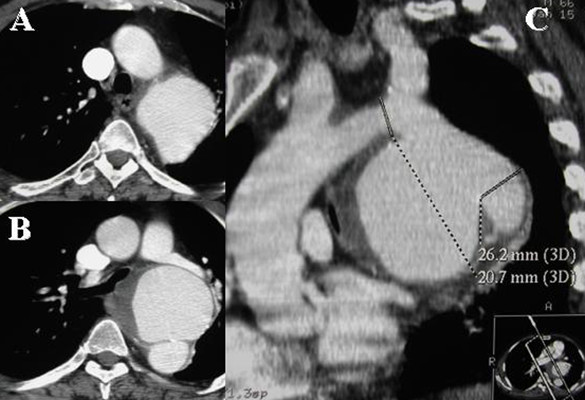
Preoperative CT-A shows an 8-cm saccular aneurysm of the distal arch (A, B); MIP reconstructions shows the involvement of the LSA (C).

Spiral CT or CT-angiography (CT-A) of the entire thoracic-abdominal aorta was performed preoperatively in all patients to determine the anatomical feasibility for the endovascular procedure, aneurysm size, location, extension, and relationship between the aneurysm and significant aortic branches such as the left subclavian artery (LSA) and the visceral vessels (e.g celiac trunk, superior mesenteric artery, renal arteries) (Figure 1). When elective surgery was scheduled, all patients underwent spirometry, echocardiography; a cardiologist was consulted to evaluate eventual either pharmacologic stress testing or coronary angiography. CT-A of the epiaortic trunks and intracranial vessels were carried out in order to detect any abnormalities (e.g. agenesia), stenosis, patency of the anterior spinal artery, or previous ischemic areas.

All patients (or their relatives) were informed in detail about the endovascular operation and gave written consent. All procedures were performed in the theatre equipped with a movable radiolucent surgical table combined with a digital angiographic system using a ceiling-suspended C-arm (Isocentric mobile C-arm-Siemens^®^; Munich-Germany), under general anesthesia by a cardiovascular surgeon in close collaboration with an interventional radiologist.

**Figure 2:**
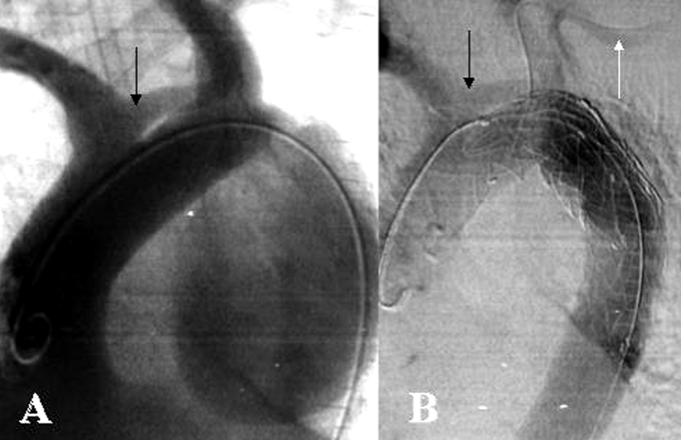
Intraoperative preliminary DSA (A) confirm the presence of a "lusoria artery" (*arrow*). Final DSA (B) confirmed the complete exclusion of the aneurysm and the patency of the left common carotid artery (*black arrow*) and the re-filling of the LSA via the ipsilateral LVA (*white arrow*).

All patients received short-term prophylactic antibiotic therapy (generally, vancomycin 2 gr b.i.d.) and heparinization intraoperatively. All patients were prepared and draped also for potential conversion to thoracotomy if it became necessary. The patients were initially positioned on their backs with the left shoulder slightly raised. Cardiopulmonary bypass (CPB) and a cell-saver (Compact-Dideco^®^-Modena, Italy) were eventually available. Prophylactic use of cerebrospinal fluid (CSF) drainage to prevent spinal cord ischemia was not used. Surgical exposure included the common femoral artery in 42 cases [monolaterally (n = 39) and bilaterally (n = 3)], an extra-peritoneal approach for the external iliac artery (n = 3), a 10-mm Dacron conduit end-to-side onto the common iliac artery (n = 1), and transperitoneal approach (n = 1) during an explorative laparptomy to repair associated multiple liver fractures in a polytraumatized patients who sustained traumatic aortic injury. Once we gained the remote access, administration of 2500 IU heparin (Epsoclar^®^-Biologici Italia Laboratories; Novate Milanese-IT) was followed by insertion of the SG system. Calibrated angiographic pigtail catheter (Cook Inc.^®^-Bloomington; IN-USA) was placed in the ascending aorta, followed by a digital subtraction angiography (DSA) to confirm the beginning of the aneurysm, to mark the LSA (e.g. a 5F catheter was preliminary advanced through a percutaneous left brachial artery access), and to determine the final landing zone. Devices were then positioned with confirmatory DSA before deployment, to enable precise positioning. Dimensions of SG were determined on the basis of contrast-enhanced spiral CT images and angiographic images, and were oversized by 10% to 20%, depending on the type of pathology; if multiple SGs wee needed, implantation began with the distal device first and then continued cephalad to the proximal end of the thoracic lesion. A complete overlap of at least 2 cm was used, with more overlap for severely angulated segments. Just before the device was released, systolic arterial blood pressure was lowered by nitro-prusside or B-blocker. Once deployed, SG were assessed with a combination of DSA and TEE to identify the presence of endoleak, evaluate the degree of device apposition, and LSA revascularization from the ipsilateral vertebral artery (Figure 2). An inflatable balloon was eventually used to model the SG to the aortic wall.

Several types of SG were used: Excluder/TAG [W.L. Gore & ass.^®^-Flagstaff, AZ-USA (n = 24)], TX-1/TX-2 [Cook Inc^®^-Bloomington, IN-USA (n = 12)], or Talent/Valiant [Medtronic Inc^®^-Minneapolis, MN-USA (n = 11)] straight tube devices. Simultaneous endovascular procedures included AAA exclusion (n = 4), axillary artery stenting (n = 3): in the former patients, both the thoracic and abdominal lesions were successfully excluded. Abdominal aortic SGs used were as following: Zenith [Cook Inc^®^-Bloomington, IN-USA (n = 3)] or Excluder [W.L. Gore & ass.^®^-Flagstaff, AZ-USA (n = 1)].

Patients were not routinely admitted to the surgical intensive care unit postoperatively. A complete CT scan was performed before the discharge in all patients to confirm complete sealing of the aneurysm and identify any endoleaks. A clinical neurological investigation was carried out in case of neurological complications, with additional diagnostics imaging if required.

**Figure 3:**
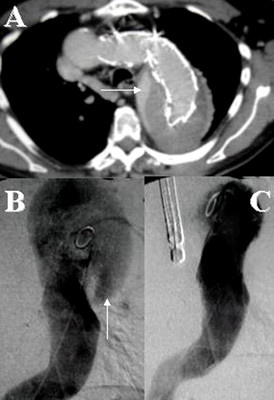
Follow-up CT-A showed late type Ib endoleak (*arrow*) after SG treatment of a type B dissecting aneurysm (A). Preliminary DSA (B) confirmed the re-perfusion of the false channel through a distal re-entry tear (*arrow*): additional SG was used to seal the aneurysm as confirmed by final DSA (C) with TEE control.

Technical success rate was defined as successful deployment of the SG without perigraft endoleak. Endoleak was classified as primary (diagnosed within 30 days of endovascular repair) or secondary (diagnosed more than 30 days after intervention) (Figure 3). All complications occurring while the patient remained in the hospital or later, were recorded. A >20% rise in renal parameters was considered indication of renal complications. Mortality rates were calculated at three intervals: during the in-hospital stay (perioperative), 30-days after the operation (early), and in follow-up (late).

The follow-up protocol featured spiral-CT or CT-A, plus conventional 4-view chest X-rays to detect changes in SG configuration at 1, 4 and 12 months after intervention, and then annually. All films were reviewed by both an attending radiologist and an attending surgeon. During the follow-up, DSA control was performed in patients with persistent endoleak, stent migration, or aneurysm sac enlargement.

## Results

In all cases, the SG was placed successfully; neither procedures aborted because of access difficulty, nor conversion to standard open repair was necessary. The median delay of the emergency procedure for traumatic injury from the original accident was 95 ± 19 minutes (range 60–360), whereas the overall median delay for the urgent procedures was 108 ± 27 minutes (range 60–1440). Overall, the mean duration of the procedure was 119 ± 75 minutes (range 45–240). Median blood loss was 254 mL (range 50–1200 mL). The average total amount of contrast was 118 ± 50 mL (range 50–240). A total of 67 SGs were implanted: 33 patients received a single SG, 10 patients two SGs, and 4 patients three SGs. The mean length of the aorta covered by the SGs was 192 ± 21 mm (range 100–360), whereas mean SG diameter was 35 ± 4 mm (range 26–42). The LSA was over-stented in 17 cases.

### Mortality

overall 30-day operative mortality was 6.4%, 3.1% in those undergoing elective treatment and 13.3% in those undergoing emergency treatment. In the elective group a cause of death was an hemorrhagic shock due to a secondary rupture owing to an undetected endoleak, 6 days after the procedure. In the emergency patients, we experienced hemorrhagic shock (n = 1) due to rupture of a complicated type B dissection, and a sudden irreversible cardiac failure (n = 1). During follow-up (mean 18 months, range 2–60), two further patients died. The causes of death were acute myocardial infarction (n = 1), and acute respiratory failure (n = 1); mean interval was 11.6-months (range 48-days-32-months). Overall, at 1, 3 and 5 years, survival for elective and emergency cases were 89.3%, 83.3% and 79%, respectively.

### Neurological sequelae

there was no paraparesis or paraplegia due to ischemia of the spinal cord. Four patients (8.5%) developed cerebrovascular events after the operation: we observed 2 cases of transient ischemic events (TIA); and 2 strokes that occurred 19 days after the intervention in a 62-years old male patient with a zone 3 type B dissecting aneurysm and was caused by recurrent peaks of hypertension that did not respond to pharmacological therapy, and 2 days after the intervention in a 79-tears male patient with a zone 2 aortic arch aneurysm. Patients with TIA did not developed definitive neurological deficits.

### Endoleaks

overall, endoleak rate was 14%. Conversion to open surgery was never required. Early endoleaks were detected before discharge in 2 patients: one type 1a endoleak and 1 type 2 endoleak from the LSA. The last one was successfully excluded by coils embolization at the origin of the LSA. One type 1a endoleak developed after caudal migration of the SG, leaded to secondary rupture and finally death. Late endoleaks occurred in 3 patients. We observed 1 type 1b endoleak that was treated by additional SG on postoperative month 6, and two type 2 leaks from the LSA (n = 1) that was sealed by coils embolization, and from an intercostal artery (n = 1) that is still under survey with no evidence of sac growth. New onset of endoleaks, at later follow up, were not observed.

### Overstented supra-aortic arteries

the LSA was fully covered in 17 patients (36%). None of the patients developed an upper arm ischemia. In 4 cases in which the origin of the left common carotid artery (LCA) was covered with a SG, a carotid-carotid PTFE bypass was performed before stent-grafting. Preventive bypass procedure to the LSA was performed in one case only in a 79-years old male patient with a zone 1 arch aneurysm; the preoperative CT-A revealed the occlusion of the right vertebral artery, hence being at high risk of perioperative cerebrovascular accident we carried out an extra-anatomical bypass to the LSA in addition to the carotid-carotid bypass, before the SG procedure. Unfortunately, the patient developed a major stroke (left part plus posterior) two days after the intervention. In the remainder cases, left upper arm ischemia did not develop, and retrograde flow through the ipsilateral vertebral artery (LVA) was demonstrated by final DSA and postoperative duplex examination. Retrograde reflux leaks from the subclavian artery into the aorta occurred in 2 cases and were sealed by coils.

### Other complications

overall, the most common complication was pneumonia (n = 8), followed by arrhythmia [atrial fibrillation (n = 3) and atrial flutter (n = 1]). Six patients (13%) developed renal impairment [(early (n = 3), late (n = 3)]. Temporary hemofiltration or dialysis was necessary in 2 patients. Two patients demonstrated new, clinically silent splenic infarctions on the postoperative CT but did not develop abscess or subsequent infectious complications; both lesions resolvedcompletely. Colic ischemia developed in a 74-year male patient on day twenty-seventh, postoperatively and required an Hartman intervention: he did well and was discharged without further complication. Among minor complications, the most frequent was post-implant syndrome (PIS) that developed in 7 patients (15%). No patients had retrograde acute ascending thoracic dissections. Thoracic lesion shrinkage, defined as a sac reduction of 5 mm at least after 12-months CT-A follow-up control, was observed in 22 patients (47%).

## Discussion

Mortality is the most important criterion in appraising the results of TAA treatment. Conventional intervention carries a mortality rate that ranges between 3% and 26%, up to 75% for emergency repair [10,11]. In their largest series, Stanford equipe reported a perioperative mortality of 9% [12]. The 6% thirty-day mortality we attained with endovascular repair, is similar to previous reports of procedure-related mortality in patients undergoing endovascular treatment of TAA and dissections and the benefits of the intraluminal approach are apparent [2,4,7,9]. Moreover, the 13% thirty-day mortality in our emergency cases are quite encouraging: considering that all the procedures were performed in high-risk patients, we believe that this early mortality rate is acceptable. It is interesting to note that patients with TAD who required emergent treatment had a procedure related mortality lower than reported, although most of the patients had active bleeding at presentation. This result suggest that, even among patients with the highest risk, endovascular repair can be undertaken with acceptable mortality. Given the high mortality in patients who undergo emergent open repair, these patients would seem to benefit most from endovascular treatment. Indeed, all the late deaths in our cohort were not related to the SG procedure; in order to reduce mortality and morbidity rates, careful selection criteria must be followed when treating patients endovascularly: using objective criteria could help in selecting patients for endovascular treatment [13].

Surgeons have often focused on death, bleeding, and paraplegia as the major adverse outcomes of thoracic aortic surgery [1,2,4-6,12]. Stroke may not be adequately appreciated as a common sequel of descending aortic operations. Most clinical reports on aortic surgical procedures simply state an overall incidence of stroke without specific analysis of this complication [14,15]. In our series, stroke was found to be disturbingly frequent after aortic surgery. In multivariable analysis, a number of risk factors were found to be predictive of stroke for open surgery: these included a history of diabetes mellitus (possibly caused by chronic damage to the cerebral microvasculature), and emergency surgery; in our experience, all but one cerebrovascular accidents were embolic-technically related, and appeared to be caused by the manipulation of wires and catheters in an atherosclerotic aortic arch [14]. Thus, meticulous technique must be used during device deployment to avoid atheroembolic cerebrovascular events.

Spinal cord ischemia and postoperative neurologic deficit with surgical repair of degenerative aneurysms and thoraco-abdominal aneurysms are believed to be multifactorial in etiology. Their occurrence is influenced by the duration and severity of the ischemic insult, the neuronal metabolic rate during this ischemic period, the postoperative hypotension, and a subsequent reperfusion injury [2,11,16,17]. In-addition, the extent of the aneurysm involvement with the thoracic aorta has been shown to influence the expected rate of neurologic deficit after open thoracic aortic aneurysm and thoraco-abdominal repair [16]. Despite many improvements in the technique and adjuncts, the rates of paralysis in conventional open surgery range from 1.5% to 19% in high-volume centers [10,11,16-19]. However, since a large number of intercostal arteries are acutely occluded even in endovascular repair, especially when long SG are implanted (a maximum of 360 mm in our patients), paralysis can still occur. In our own patients, there has been no case of paralysis up to now, despite the average length of the "stented" thoracic aorta was 195 mm, which means that 4 to 5 pairs of intercostal arteries were occluded at least. The aortic segment from T7 to T11, regarded as particularly sensitive, was covered with SG in 10 of our patients.

Because of the absence of aortic cross-clamping and reperfusion, endovascular SG repair of the descending thoracic aorta may lower the incidence of spinal cord ischemia. Those cases with TAA stent-grafting with concomitant open AAA repair also undergo a period of aortic cross-clamping, potentially causing an even more severe initial spinal cord ischemia as well as adding an element of reperfusion injury to the ischemic insult, being associated with an increased risk of paraplegia, since the absence of the normal lumbar or ilio-lumbar arteries sacrificed by AAA surgery may place these candidates at an increased risk for spinal cord ischemia [11,20-23]. We noted no paraplegia or paraparesis, although the distal thoracic aorta was stented in eight patients with previous abdominal aortic surgery, and four patients had concomitant endovascular aortic multilevel disease.

Although in the initial steps of previous published studies, endoleaks in the thorax seemed markedly less significant than of aneurysms of the infrarenal aorta, it soon became obvious that endoleaks after thoracic endovascular therapy are a serious complication [1,4,7,8,12]. Degenerative disease and atherosclerotic aneurysm formation of the thoracic aorta are diffuse and progressive in character, often involving the entire thoracic aorta, but changes within the proximal and distal neck caused by disease progression have been reported causing type 1 endoleak [7]. Neck dilatation after endovascular aneurysm repair has previously been recognized in 2 patients by a group at Stanford University. In our series 2 patients displayed neck dilatation [12]. Our policy in treating type Ib endoleaks was to implant a cuff at the endoleak site. These late complications show the necessity of mandatory life-long follow-up. Finally, disease progression proximally or distally, especially in patients with diffuse degenerative aneurysms, may result in slow enlargement of the landing zones, loss of fixation, and sac pressurization [2,7]. Isolated cases of secondary rupture after SG have been described in many reports. In all of these cases, technical errors of untreated primary or secondary endoleaks could be incriminated. In this series post-procedural rupture was observed in one patient. Multiple factors may have contributed to these late endoleaks. The association between action of vector forces in SG and the often severe tortuosity in the thoracic aorta might induce even stronger vector forces after aneuryms repair.

Renal failure (RF) has been reported as one of the leading cause of postopretaive death following conventional descending aneurysm and thoraco-abdominal repair; also endovascular repair involved new potential risks specific to the procedure, which may be associated with a substantial rate of postoperative RF [23]: the incidence of long-term RF following endovascular aortic aneurysm exclusion is poorly documented in the literature; the incidence of postoperative RF ranged between 2.5% and 6.6% in previous reported series [24]. In the present study, transient perioperative RF occurred in 13% of patients; moreover, it has been noted a trend toward this being a more common event in those patients with pre-existing RF [18,24,25]. We identified a 17% prevalence of preoperative RF in our own population undergoing endovascular repair. Our incidence of renal complications is similar than rates reported by others [24]. Most likely the etiology is multifactorial, resulting from a combination of mechanical, atheroembolic and contrast related contributors. In particular, the relationship between contrast type and volume and renal function has been established [25]. Contrast induced renal insufficiency is reversible, yet in our patients, the noted creatinine rise was permanent in only 1 case of those patients who had deterioration of renal function: this suggests factors other than contrast exposure alone as contributory, suggesting atheroembolic sequelae. Strategies may be adopted to minimize the likelihood of adverse renal effects associated with aortic SG at all stages of evaluation and intervention. While our routine practice involves the use of non-ionic contrast agents in association with vigorous hydration and use of osmotic diuretics, we have greatly reduced our reliance upon angiography employing iodinated contrast agents in our patients with RF.

## Conclusion

Although this report is a retrospective and not comparative analysis of thoracic aortic repair, the combined minor and major morbidity rate was lower than previous reported to results of either electively and emergency performed conventional repair.

Despite the favorability of these findings, complications unique to endovascular repair of descending thoracic aortic lesions (eg, RF, stroke, leakage) did occur. These complications in part reflect the results of an evolving technology that involves a requisite learning curve and developmental issues. A reduction in these method-related complications can in all probability be achieved only by improved SG and optimal patient selection and matching.

In addition to the usual concerns of death, bleeding, and paraplegia, surgeons must consider stroke heavily when deciding to operate on the thoracic aorta. Meticulous technique must be used during device deployment to avoid atheroembolic cerebrovascular events.

Renal insufficiency is present in a large percentage of patients being considered for endovascular aneurysm repair. It should not be considered a contraindication to the evaluation or management of these patients provided that care is taken to pursue a strategy that minimizes the risk of renal injury.

## Competing interests

The author(s) declare that they have no competing interests.

## Authors' contributions

GP: study design; data collection; performed the statistical analysis.

MT: critical analysis

CL: data collection

NR: critical analysis

RC: critical analysis

PC: critical and statistical analyses

All authors read and approved the final manuscript.

## References

[B1] Heijmen RH, Deblier IG, Moll FL, Dossche KM, van den Berg JC, Overtoom TT, Ernst SM, Schepens MA (2002). Endovascular stent-grafting for descending thoracic aortic aneurysms. Eur J Cardiothorac Surg.

[B2] Czerny M, Cejna M, Hutschala D, Fleck T, Holzenbein T, Schoder M, Lammer J, Zimpfer D, Ehrlich M, Wolner E, Grabenwoger M (2004). Stent-graft placement in atherosclerotic descending thoracic aortic aneurysms: midterm results. J Endovasc Ther.

[B3] Brandt M, Hussel K, Walluscheck KP, Muller-Hulsbeck S, Jahnke T, Rahimi A, Cremer J (2004). Stent-graft repair versus open surgery for the descending aorta: a case-control study. J Endovasc Ther.

[B4] Neuhauser B, Perkmann R, Greiner A, Steingruber I, Tauscher T, Jaschke W, Fraedrich G, Czermak BV (2004). Mid-term results after endovascular repair of the atherosclerotic descending thoracic aortic aneurysm. Eur J Vasc Endovasc Surg.

[B5] Bell RE, Taylor PR, Aukett M, Sabharwal T, Reidy JF (2003). Mid-term results for second-generation thoracic stent-grafts. Br J Surg.

[B6] Cambria RP, Brewster DC, Lauterbach SR, Kaufman JL, Geller S, Fan CM, Greenfield A, Hilgenberg A, Clouse WD (2002). Evolving experience with thoracic aortic stent graft repair. J Vasc Surg.

[B7] Scharrer-Pamler R, Kotsis T, Kapfer X, Gorich J, Orend KH, Sunder-Plassmann L (2003). Complications after endovascular treatment of thoracic aortic aneurysms. J Endovasc Ther.

[B8] Hansen CJ, Bui H, Donayre CE, Aziz I, Kim B, Kopchok G, Walot I, Lee J, Lippmann M, White RA (2004). Complications of endovascular repair of high-risk and emergent descending thoracic aortic aneurysms and dissections. J Vasc Surg.

[B9] Ellozy SH, Carroccio A, Minor M, Jacobs T, Chae K, Cha A, Agarwal G, Goldstein B, Morrissey N, Spielvogel D, Lookstein RA, Teodorescu V, Hollier LH, Marin ML (2003). Challenges of endovascular tube graft repair of thoracic aortic aneurysm: midterm follow-up and lessons learned. J Vasc Surg.

[B10] Schepens M, Dossche K, Morshuis W, Heijmen R, van Dongen E, Ter Beek H, Kelder H, Boezeman E (2004). Introduction of adjuncts and their influence on changing results in 402 consecutive thoracoabdominal aortic aneurysm repairs. Eur J Cardiothorac Surg.

[B11] Biglioli P, Spirito R, Porqueddu M, Agrifoglio M, Pompilio G, Parolari A, Dainese L, Sisillo (1999). Quick, simple clamping technique in descending thoracic aortic aneurysm repair. Ann Thorac Surg.

[B12] Demers P, Miller DC, Mitchell RS, Kee ST, Sze D, Razavi MK, Dake MD (2004). Midterm results of endovascular repair of descending thoracic aortic aneurysms with first-generation stent-grafts. J Thor Cardiovasc Surg.

[B13] Rodrigues Alves CM, da Fonseca JH, de Souza JA, Camargo Carvalho AC, Buffolo E (2002). Endovascular treatment of thoracic disease: patient selection and a proposal of a risk score. Ann Thorac Surg.

[B14] Bachet J, Guilmet D, Goudot B, Dreyfus GD, Delentdecker P, Brodaty D, Dubois C (1999). Antegrade cerebral perfusion with cold blood: a 13-year experience. Ann Thorac Surg.

[B15] Di Bartolomeo R, Di Eusanio M, Pacini D, Pagliaro M, Savini C, Nocchi A, Pierangeli A (2001). Antegrade selective cerebral perfusion during surgery of the thoracic aorta: risk analysis. Eur J Cardiothorac Surg.

[B16] Coselli JS, Lemaire SA, Koksoy C, Schmittling ZC, Curling PE (2002). Cerebrospinal fluid drainage reduces paraplegia after thoracoabdominal aortic aneurysm repair: results of a randomized clinical trial. J Vasc Surg.

[B17] Quinones-Baldrich WJ (2004). Descending thoracic and thoracoabdominal aortic aneurysm repair: 15-year results using a uniform approach. Ann Vasc Surg.

[B18] Cambria RP, Clouse WD, Davison JK, Dunn PF, Corey M, Dorer D (2002). Thoracoabdominal aneurysm repair: results with 337 operations performed over a 15-year interval. Ann Surg.

[B19] Safi HJ, Miller CC, Huynh TT, Estrera AL, Porat EE, Winnerkvist AN, Allen BS, Hassoun HT, Moore FA (2003). Distal aortic perfusion and cerebrospinal fluid drainage for thoracoabdominal and descending thoracic aortic repair: ten years of organ protection. Ann Surg.

[B20] Moon MR, Mitchell RS, Dake MD, Zarins CK, Fann JI, Miller DC (1997). Simultaneous abdominal aortic replacement and thoracic stent-graft placement for multilevel aortic disease. J Vasc Surg.

[B21] Meguid AA, Bove PG, Long GW, Kirsch MJ, Bendick PJ, Zelenock GB (2002). Simultaneous stent-graft repair of thoracic and infrarenal abdominal aortic aneurysms. J Endovasc Ther.

[B22] Melissano G, Civilini E, Bertoglio L, Setacci F, Chiesa R (2005). Endovascular treatment of aortic arch aneurysms. Eur J Vasc Endovasc Surg.

[B23] Carpenter JP, Fairman RM, Barker CF, Golden MA, Velazquez OC, Mitchell ME, Baum RA (2001). Endovascular AAA repair in patients with renal insufficiency: strategies for reducing adverse renal events. Cardiovasc Surg.

[B24] Bakris GL, Lass NA, Glock D (1999). Renal hemodynamics in contrast media induced renal dysfunction. A role for dopamine-1 receptors. Kidney Int.

[B25] Topel M, Giet MVD, Schwarzfeld C, Laufer U, Liermann D, Zidek W (2000). Prevention of radiographic contrast agent induced reductions in renal function by acetylcysteine. N Engl J Med.

